# A Framework for Understanding the Role of Psychological Processes in Disease Development, Maintenance, and Treatment: The 3P-Disease Model

**DOI:** 10.3389/fpsyg.2019.02498

**Published:** 2019-11-20

**Authors:** Casey D. Wright, Alaina G. Tiani, Amber L. Billingsley, Shari A. Steinman, Kevin T. Larkin, Daniel W. McNeil

**Affiliations:** ^1^Department of Psychology, West Virginia University, Morgantown, WV, United States; ^2^Department of Dental Practice and Rural Health, School of Dentistry, West Virginia University, Morgantown, WV, United States

**Keywords:** health psychology, disease etiology, biopsychosocial, heart disease, pain, psychogastroenterology, behavioral dentistry, behavioral medicine

## Abstract

Health psychology is multidisciplinary, with researchers, practitioners, and policy makers finding themselves needing at least some level of competency in a variety of areas from psychology to physiology, public health, and others. Given this multidisciplinary ontology, prior attempts have been made to establish a framework for understanding the role of biological, psychological, and socio-environmental constructs in disease development, maintenance, and treatment. Other models, however, do not explain *how* factors may interact and develop over time. The aim here was to apply and adapt the 3P model, originally developed and used in the treatment of insomnia, to couch the biopsychosocial model in a way that explains how diseases develop, are maintained, and can be treated. This paper outlines the role of predisposing, precipitating, and perpetuating factors in disease states and conditions (the 3Ps) and provides examples of how this model may be adapted and applied to a number of health-related diseases or disorders including chronic pain, gastrointestinal disorders, oral disease, and heart disease. The 3P framework can aid in facilitating a multidisciplinary, theoretical approach and way of conceptualizing the study and treatment of diseases in the future.

## Introduction

Scientific and technological advances in 1969 led to “giant leaps for mankind,” including the publication of a paper applying psychological principles to health services (Schofield, 1969). Over the next several years, the American Psychological Association (APA) established a task force to explicate the role of behavior and psychology in health-related processes, systems, and diseases (APA Task Force on Health Research, 1976). As a result, health psychology and behavioral medicine were established as unique fields of study (Wallston, 1996). Contemporary uses often use the terms health psychology and behavioral medicine interchangeably.

Since their early roots, health psychology, and behavioral medicine, as fields of study and areas of practice, have grown in both breadth and depth. Whether it is the latest popular press article about the gut-brain axis (e.g., Abdel-Haq et al., 2019), mindfulness (e.g., Dahlgaard et al., 2019), or dental anxiety (e.g., Zinke et al., 2019), psychological processes have a demonstrable role in health and well-being (and vice-versa). Indeed, the field of health psychology is a burgeoning area of research with direct clinical applications. Ontologically, health psychology researchers, and practitioners find themselves required to attain some level of expertise (or at minimum be conversant) in not only psychology, but also immunology, microbiology, physiology, health policy, and more. Thus, health psychology contributes to the interdisciplinary effort, and a general-yet-targeted framework is needed to help conceptualize and guide future work in understanding the specific role of psychological constructs (e.g., behaviors, cognitions) in disease development, maintenance, and treatment.

A number of theories and models have been put forth to describe various components of medicine and health, including the biopsychosocial model which emphasizes the role of psychological and social factors in addition to biological factors in health and disease (Engel, 1977; Borrell-Carrió et al., 2004). The biopsychosocial model was developed as a response, in part, to biological reductionism (Engel, 1977) and has played an integral role across multiple disciplines including health psychology and behavioral medicine (Taylor, 2018). While the biopsychosocial model was important in communicating the importance of considering a patient as a whole being, a number of weaknesses with the model have been identified over the years (McLaren, 1998; Ghaemi, 2009; Benning, 2015). Well-argued elsewhere, the biopsychosocial model has been criticized for being non-specific or eclectic (Ghaemi, 2009), and not sensitive enough to individual differences and needs (Benning, 2015). Some have even argued that although the biopsychosocial model has been around for over 40 years, its influence is not as widespread as was anticipated in affecting how health and disease are conceptualized or managed today (Kontos, 2011). This could be, in part, because the biopsychosocial model lacks a framework for understanding *how* biological, psychological, and socio-environmental factors may contribute at each stage of disease development, in maintaining disease, or in the treatment of disease.

While the biopsychosocial model may help in stating that multiple disciplines contribute to health and disease, more theoretical work is needed to conceptualize and explicate *how* interdisciplinary factors – including psychological and behavioral ones – contribute to the etiology, maintenance, and treatment of disease states and conditions. That is, more conceptual work needs to be done to explain *how* biological, sociological, and psychological factors interact, rather than simply stating that they *do* interact. The application and combination of another well-known model (i.e., the 3P model) outlined here provides a more robust, and importantly, a time-inclusive argument of how different factors may manifest and subsequently accumulate to propel an individual up the “ladder” and over the “threshold” of disease manifestation. As will be argued, what contributes to or often maintains disease states can be multi-faceted, and can include maladaptive behavioral, cognitive, and emotional factors. Others have put forth similar ideas (Bolton, 2014) including using some of the same terminology (“4Ps”) in proposing case conceptualization in this manner. What lacks, however, is the practical and tangible application of such a model to specific diseases and future directions.

The purpose of this paper is to adapt, utilize, and apply a model first outlined for insomnia to provide a comprehensive framework by which researchers and providers can understand the interdisciplinary nature of disease development, maintenance, or interventions, and specifically how psychology fits within such a system. Clarifying and providing such a framework in disease processes could aid in couching future work that can lead to more meaningful interventions by psychologists or other healthcare providers in understanding and facilitating the prevention, mitigation, or treatment of any given disease state or condition.

### Insomnia and the 3P Model

Insomnia is a unique disorder affecting many individuals and is characterized by a persistent issue with initiating and maintaining sleep (Bhaskar et al., 2016; Centers for Disease Control and Prevention, 2019). Interestingly, a behavioral treatment is considered a first-line, evidence-based practice for helping patients overcome insomnia (Qaseem et al., 2016; Riemann et al., 2017). Cognitive behavioral treatment of insomnia (CBT-I; Perlis et al., 2005) is a manualized treatment with robust evidence for aiding individuals with their sleep problems. Changes in sleep patterns fostered by CBT-I also tend to be more durable over time than alternative treatments such as medications (Perlis et al., 2005).

**FIGURE 1:**
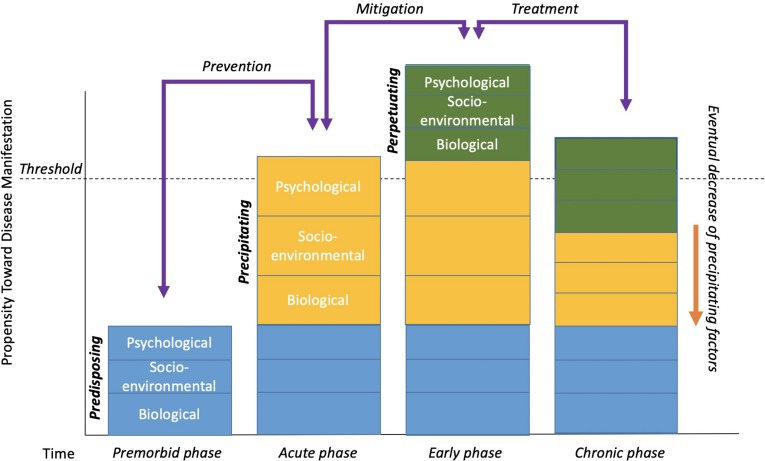
The 3P-Disease Model. The *x*-axis represents time and the *y*-axis represents the propensity toward disease manifestation. Reuse/adapted from The Psychiatric Clinics of North America, volume 10, A.J. Spielman, L.S. Caruso, and P.B. Glovinsky, A behavioral perspective on insomnia treatment, 541–555, Copyright (1987), with permission from Elsevier.

Cognitive behavioral treatment of insomnia is based on a behavioral model first introduced by Spielman et al. (1987) and is colloquially known as “the 3P Model.” The three Ps – predisposing, precipitating, and perpetuating factors – all contribute to the development and maintenance of chronic insomnia. Figure 1 displays an adapted version of the 3P model as originally depicted by Spielman et al. (1987).

#### Predisposing Factors

Contributing factors that are long-standing or lasting are characterized as predisposing. Certain genes and family histories, or even long-standing environmental, social, or psychological factors could all be considered predisposing factors. In the case of insomnia, an individual may simply be genetically predisposed or perhaps have a biological disposition toward being hyper-aroused that contribute to difficulty falling asleep, staying asleep, or waking too early. Importantly, predisposing factors are not synonymous with the “bio” in the biopsychosocial model. Biological factors may contribute significantly to disease, perhaps, but are not sufficient in causing, maintaining, or even treating some diseases by themselves. That is, predisposing factors occur in the premorbid phase of disease (Perlis et al., 2005) and can be socio-enviornmental or psychological in nature as displayed in Figure 1. For example, an individual may live with a partner whose work schedule or own sleep habits lead to disrupted sleep patterns. Likewise, the ability to afford a home away from train tracks, or other long-standing environmental factors may be difficult to change. From a psychological perspective, individuals may have a more trait-like cognitive predisposition to seeing the world as a more negative place or to worry more, thus being easily awoken.

#### Precipitating Factors

More often than not, predisposing factors alone do not lead to dramatic sleep problems or to chronic insomnia. Most likely, there are specific events or situations that precipitate or initially lead to the onset of insomnia symptoms. For example, having or adopting a new baby can lead to major sleep disruptions for many new parents. The stress associated with losing a job, grieving over the loss of a loved one, ending a long-term relationship, and many other situations may lead to disrupted and poor sleep (e.g., fewer hours slept, being awoken in the middle of the night, tossing, and turning, waking early). In Figure 1, precipitating factors are usually what initially “push” someone over the threshold of clinically significant insomnia in its acute phase.

It also is important to mention here that precipitating factors need not be single- or one-time events. Precipitating factors can be multiple or recurrent events or situations that build over time to contribute to disease development or manifestation (e.g., having a new baby and getting in a car accident). Similarly, precipitating factors could be environmental changes (e.g., job change or loss) that precipitate the condition emerging into the clinical “threshold” (Perlis et al., 2005). Psychological factors can precipitate sleep problems as well, such as when a loved one dies and the individual grieves, leading them to have more or less sleep drive.

#### Perpetuating Factors

Typically, acute stressors and precipitating factors will resolve over time in the early phase of disease development or maintenance (Perlis et al., 2005). That is, things can get better on their own, or be mitigated by natural adjustment processes. Babies eventually learn to sleep on a schedule, individuals gain new employment, people progress through the grief, and mourning process, and so on. In some cases, however, during the precipitating times, individuals begin to engage in maladaptive behavior and/or thinking patterns in an attempt to compensate for, or cope with, the difficult or stressful times. For example, someone experiencing lack of sleep during the newborn phase of parenthood may begin napping during the daytime, drinking more caffeine than normal, sleeping in every now and again, etc. They also may engage in negative thinking styles such as catastrophizing (e.g., If I don’t fall asleep now, I’m going to do an awful job at work tomorrow and might end up getting fired), or all-or-nothing thinking (e.g., If I don’t fall asleep by 10:30, I’m probably going to be up all night). Over time, these compensatory acts that were intended to be helpful often become habits and are actually counterproductive to good sleep. These behaviors and thinking patterns are considered *perpetuating* factors. As is displayed in Figure 1, perpetuating factors are what typically maintain certain conditions into chronicity even after the natural course of a precipitating factor(s) subside.

## The 3P-Disease Model

### Importance of Thoughts, Feelings, and Behavior in Disease

The 3P model has broad implications beyond insomnia and can aid in explaining the role of behavior, cognition, and emotions to a multitude of presenting issues. Applications for the 3P model in the development and maintenance of disease in any setting are abundant. To demonstrate only a few potential applications to a variety of diseases, applications of the 3P model to chronic pain, gastrointestinal disorders, oral, and dental disease, as well as heart disease are postulated. These specific diseases are presented to explicate the utility and applicability of the 3P model to a wide range of seemingly unrelated diseases, but also to demonstrate that many of predisposing, precipitating, and perpetuating factors underlying these diseases are functionally similar in nature (e.g., health behaviors, coping mechanisms, etc.). These examples were chosen because they include some of the more prevalent or burdensome diseases faced by society and because of the authors’ familiarity with applying the 3P model to these diseases in research and practice. This work should not preclude others from adding additional insight into other diseases as well. As aforementioned, beyond demonstrating the *importance* of psychological factors, the 3P model provides a conceptual framework for understanding *how* psychological factors may be playing a role in disease development and maintenance.

**FIGURE 2:**
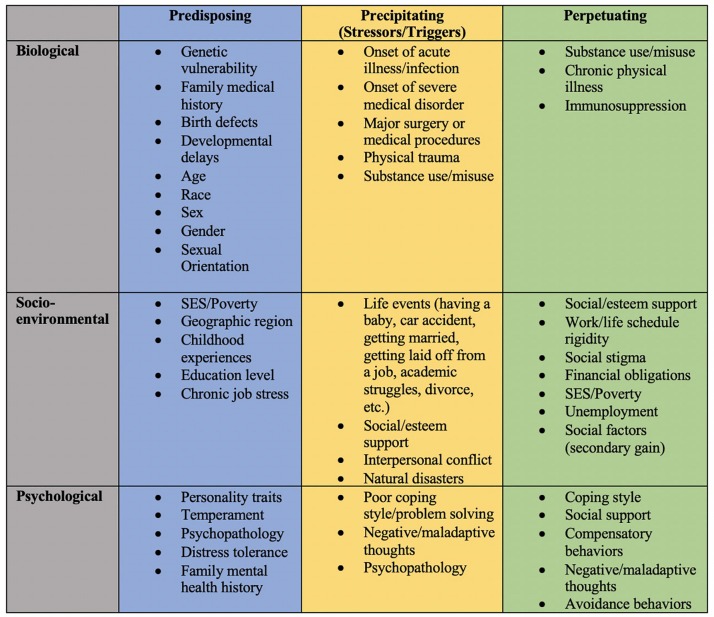
Summary of potential predisposing, precipitating, and perpetuating factors across biopsychosocial domains.

Depending on the disease in question, biological, socio-environmental factors, behavioral, and psychological factors may function as one of many causal or maintaining factors of disease states. Examples of each of these situations will be provided below. The primary aim of this paper is to demonstrate the utility of the 3P model, an enhanced perspective of the biopsychosocial model that includes how predisposing, precipitating, and perpetuating factors contribute to disease development, maintenance, and treatment. Doing so will highlight the equally needed attention in disease research and treatment of biological, socio-environmental, and especially psychological factors. This reconceptualization and new perspective will additionally aid in identifying how physicians/dentists, psychologists, and researchers or professionals from all disciplines can work together to better elucidate a variety of disease etiologies and establish innovative treatments. Examples of possible applications of the 3P model below are just a sampling of how diseases may be re-conceptualized to help facilitate the aforementioned aims. Figure 2 summarizes some of the common 3P factors across each of the biopsychosocial domains.

### Examples of 3P-Health Model Applications to Specific Diseases

#### Chronic Pain

In recent years, problems with chronic pain have greatly increased. Chronic pain can cause high levels of distress and often leads to significant functional impairment in a variety of domains (e.g., social and occupational). The vast economic impact of chronic pain includes direct costs due to increased utilization of medical services as well as indirect costs due to lost wages and decreased productivity. In fact, it is estimated that the United States lost up to $635 billion in 2010 due to costs associated with chronic pain, and given the worsening of the opioid epidemic in recent years, it is likely that these estimates are higher today (Institute of Medicine, 2011). Importantly, the economic burden of pain is higher than that of cancer, diabetes, and heart disease, all of which are significant public health problems themselves (Institute of Medicine, 2011).

The increase in the prevalence of chronic pain is closely tied to a worsening of the opioid epidemic. Opioids have been prescribed at record levels, with rural and lower socioeconomic status (SES) areas being some of the most impacted by this growing problem (Keyes et al., 2014; Prunuske et al., 2014; Guy et al., 2017). The economic impact of the opioid epidemic is drastic, with costs in the United States estimated up to $78.5 billion in 2013 for prescription opioid use disorder and overdoses (Florence et al., 2016).

##### Predisposing factors

There are a variety of factors that may increase an individual’s predisposition to developing chronic pain. Broadly, these factors include genetic predispositions, long-standing environmental factors such as a physically demanding job, and other issues such as one’s physiological pain threshold or their ability to tolerate physical discomfort (Diatchenko et al., 2004). Consistent with the 3P model, having any one of these predisposing factors increases one’s vulnerability to developing chronic pain but is likely not sufficient on its own to push an individual past the threshold to develop chronic pain. In other words, these are simply risk factors for developing chronic pain.

Research on genetic predisposition for chronic pain syndromes is a rapidly developing, nascent area of study (Gatchel et al., 2007; James, 2017). The risk for chronic pain is considered a heritable trait with estimates of heritability up to 38.4% (McIntosh et al., 2016). Though researchers have identified some genetic links for chronic pain, much of this research has been conducted over only the past decade and the extent to which genes actually influence the development of chronic pain is still unknown. While no single genetic explanation for chronic pain exists, some hypothesized mechanisms by which it develops are through epigenetic processes and single nucleotide polymorphisms, though findings are somewhat mixed (James, 2017).

Researchers also found that having a spouse or partner with chronic pain increases an individual’s risk for developing chronic pain themselves, as does having major depressive disorder (McIntosh et al., 2016). There also are individual differences in pain threshold and tolerance that may contribute to the development of chronic pain (Diatchenko et al., 2004). Individuals who experience pain as more intense or aversive than others are likely to recognize feelings of physical discomfort more readily than those who do not experience pain as such. An individual who is more sensitive to feelings of pain may therefore be at greater risk of developing chronic pain than those whose pain threshold is much lower and who require less medical intervention to manage their pain. Pain catastrophizing is another similar predisposing factor related to the eventual development of chronic pain (Leung, 2012). Pain catastrophizing, which refers to a sense of helplessness as well as the amplification of the experience of pain due to anxious rumination, has been shown to be an independent risk factor for developing chronic pain following a total knee arthroplasty surgery (Riddle et al., 2010; Burns et al., 2015).

##### Precipitating factors

There are a variety of situations and environmental factors that will initially precipitate the development of chronic pain. Some examples of this are surgical procedures (such as knee arthroplasty surgery, discussed above), work-related injuries, car accidents, sports injuries, and other life events or stressors that lead to pain. In the acute phase immediately following these events, the afflicted individual may believe that their pain will be a temporary occurrence that will go away once the situation resolves, though importantly, beliefs about pain duration are generally not related to actual pain duration (Williams and Thorn, 1989; Williams and Keefe, 1991). For instance, they may believe that once their body physically recovers from the injury, their pain will cease. Unfortunately, since these beliefs about pain duration are not always accurate, many individuals who experienced some acute precipitating event go on to develop a chronic pain problem. Oftentimes, this can be directly attributed to perpetuating behaviors in which the individual engages in order to reduce their experience of pain.

Although the precipitating events that lead to the development of chronic pain tend to be more obvious than precipitating events that lead to other health problems, many individuals discount the important role that stress can play in the development of chronic pain (Abdallah and Geha, 2017). For example, an individual who experiences some type of stressful life event may begin to experience frequent migraines due to their high stress levels (Borsook et al., 2012) and begin engaging in unhelpful behaviors to try to manage their newfound pain. This “wear and tear” on the body due to chronic exposure to stress is referred to as allostatic load, which can increase the chance of having more pain in the future (Borsook et al., 2012). Importantly, stress and allostatic load can be conceptualized as both a predisposing and a precipitating factor.

##### Perpetuating factors

In the case of chronic pain, perpetuating factors refer to any specific behaviors that an individual does in an attempt to alleviate or distract themselves from their pain. Unfortunately, although these behaviors may improve the experience of acute pain in the moment, they often contribute to the development of chronic pain over time.

Long-term use of opioids, for example, has actually been shown to contribute to opioid-induced hyperalgesia, which is an increase in pain sensitivity that develops from chronic use of opioid medications (Ballantyne and Mao, 2003; James, 2017). Thus, although opioid medications can be used in the acute stages immediately following an injury or surgery to successfully reduce pain, continued use of these medications may actually lead to individuals becoming even more sensitive to pain, perpetuating the problem over time. Although opioids continue to be prescribed for chronic pain, there is evidence to suggest that prolonged opioid therapy may not be effective and may have negative implications for patient safety (Ballantyne and Mao, 2003). Specifically, opioid therapy for chronic pain has been linked with an increased risk for overdose, abuse, and dependence (Chou et al., 2015).

Another behavior that perpetuates pain is avoidance. When individuals are recovering from painful experiences (e.g., after surgery or following an injury), they may avoid moving the afflicted body part in an attempt to reduce pain during the recovery process (Vlaeyen et al., 1995a, b). Unfortunately, though, this avoidance can ultimately lead to more pain if the muscles or joints become stiff and underworked. Over time, this avoidance of painful experiences is negatively reinforcing because it removes fear of anticipating pain and increases the likelihood that an individual will continue to avoid painful experiences in the future. This describes the fear avoidance model (Vlaeyen and Linton, 2000), and may also be related to opioid abuse for chronic pain due to a persistent fear of experiencing pain.

A final perpetuating factor for chronic pain for some individuals could be receiving some type of benefit due to being in pain, referred to as secondary gain (Fishbain et al., 1995). Examples of secondary gain can include anything from receiving disability to having other people do things for you. Receiving benefits for being in pain is behaviorally reinforcing, and can turn acute pain into a more chronic problem.

##### Discussion of application to chronic pain

To illustrate how the 3P model explains the development of chronic pain, consider a 37-year old male who works as a mechanic. He reports that he has always had low pain tolerance and is quick to take over-the-counter pain medications following a long day at work (due to back pain from bending over to work on cars). His low pain tolerance serves as a predisposing factor. One day, he suffers a workplace injury and has to take time off to recover, which is a precipitating factor. As he recovers, he is so concerned about doing anything to exacerbate his pain that he does not engage in his prescribed physical therapy exercises and asks his spouse to do more of his chores, so he does not have to move around as much. Over time, his muscles become sore and underworked, which increases his pain, and his spouse picking up his housework becomes a nice perk that allows him to spend more time doing sedentary activities that he enjoys (e.g., watching TV). His avoidance of pain-inducing activities and requesting that his spouse complete chores for him are both perpetuating factors that turned his acute pain into a chronic problem.

Fortunately, the role of psychology in pain management is already fairly well-established and there has been an increasing need for psychosocial interventions in this area (Jensen and Turk, 2014). Pain catastrophizing, a common predisposing factor, can be targeted by psychosocial interventions, such as cognitive behavior therapy or acceptance and commitment therapy. Further, interventions to aid in stress management and targeting avoidance are common in the psychological treatment of chronic pain. Thus, it is clear how psychology can help prevent, mitigate, or treat chronic pain at all three levels of the 3P model.

#### Gastrointestinal Disease and Disorders

Expenditures on gastrointestinal diseases in the United States have been estimated around $136 billion, with over 30 million office and emergency room visits being tied back to primarily gastrointestinal complaints (e.g., abdominal pain, vomiting, and diarrhea; Peery et al., 2019). The digestive system is an essential and intricate mechanism by which food and other nutrients get absorbed by the body (National Institute of Diabetes and Digestive and Kidney Diseases, 2019). In addition to providing a mechanism by which an organism fuels their body, the gastrointestinal tract also serves as an essential factor in protecting an organism from pathogens or other outside influences that may cause disease or dysfunction (Sturgeon and Fasano, 2016). Many gastrointestinal diseases and disorders exist, some with clearer biological underpinnings (i.e., Celiac disease), and others with no clear biological etiology for the malady exists (i.e., functional gastrointestinal disorders). In any case, psychological or behavioral factors have been implicated in both structural and functional digestive diseases (Drossman et al., 1999; Häuser et al., 2010; Sato and Fukudo, 2015).

##### Predisposing factors

Prior work has argued the improbability of a single genetic factor existing that predisposes an individual to gastrointestinal disease (e.g., *GNB3*; Adam et al., 2007; Saito et al., 2010). However, consistent with the 3P conceptualization of disease development, there is ample evidence of *some* genetic risk (Adam et al., 2007). For example, it is near impossible for a patient to have Celiac disease without a variant of the *HLA-DQ2 or -DQ8* genotypes (Sturgeon and Fasano, 2016). Simply having a *DQ2 or DQ8* variant, however, does not dictate whether an individual will develop the disease. In fact, around 30% of individuals in the United States carry such a variant (Wolters and Wijmenga, 2008; Husby et al., 2012). Crohn’s disease also occurs at higher rates within families, yet no clear genetic factor has been implicated (Genetics Home Reference, 2019) and the same could be said of other gastrointestinal diseases. Suffice it to say, that typically genetic factors rarely in and of themselves cause a disease.

Genetic factors are not the only predisposing factors in gastrointestinal disease. Certainly, diet patterns can affect the digestive tract. Diet habits can be learned via various means, but familial transmission of diet or other health habits have been shown to influence overall health (Jahnke and Warshburger, 2008; Scaglioni et al., 2008). Additionally, long-standing environmental factors may be considered predisposing factors in gastrointestinal diseases. For example, if an individual resides in a location where access to healthy food choices is sparse, their diet patterns certainly will have long-term implications on health and well-being (Beaulac et al., 2009), much like in the other diseases mentioned.

##### Precipitating factors

Acute gastrointestinal distress such as diarrhea or vomiting are near-ubiquitous experiences given that most individuals face some form of viral gastroenteritis in their lifetime (Stuempfig and Seroy, 2019). Many also have issues with their gallbladder, gastrointestinal cysts, ulcers, or even food poisoning. Analogous to having a baby or losing a job in the case of insomnia, all of these events can be the result of some acute problem or disease state (e.g., eating bad food resulting in gastroenteritis, or a really intense bout of workplace stress leading to ulcers). The body and immune system also react in such a way that is considered normal when experienced acutely. That is, increased inflammation heals the affected tissue and works to kill unwanted bacteria or viruses (Segerstrom and Miller, 2004). Behaviorally, one may experience increases in disgust sensitivity (e.g., loss of appetite, things making one queasy) as an evolutionary mechanism to avoid certain foods and experiences that may prolong the disease or symptoms of the disease (Schaller and Park, 2011). For the most part, individuals deal with and overcome these ailments and it has little effect on them long-term (Schaller and Park, 2011). To reiterate, it can be healthy to have such reactions in the acute phase. Overtime, inflammation subsides, and disgust sensitivity or other psychological/behavioral factors also return to normal. For others, though, these acute and stressful times with gastrointestinal distress lead them to compensate and develop behavior, cognition, or emotions that may actually perpetuate the gastrointestinal distress to become more chronic.

##### Perpetuating factors

Many individuals with functional gastrointestinal disorders such as irritable bowel syndrome (IBS) have anxiety and fear about going to restaurants or public places (Whitehead et al., 2002; Labus et al., 2004; Roohafza et al., 2016). This could be, in part, because of negative social or health-related experiences of urgent diarrhea and worry about public embarrassment (Labus et al., 2004). Over the normal course of disease and treatment, however, especially with well-managed symptoms, individuals will still engage in behavior or thoughts about certain situations. For example, even though someone may have well-managed IBS, they may fear going with friends to a restaurant. The friends may eventually talk this person into going, but upon arriving and eating the first bite (of a safe food for them to eat), the individual “feels something” in their bowels and they begin to panic. This leads them to need to run to the restroom to void and their fears are confirmed. This hyper-visceral sensitivity (Labus et al., 2004) may have contributed in part to the biological gastrointestinal distress experienced. Others have shown the connection between anxiety and gastrointestinal distress (Häuser et al., 2010). Exact mechanisms of why this occurs are not well-understood, but the 3P conceptualization may aid in future work to explore this area in more depth.

##### Discussion of application to gastrointestinal disorders

Take into consideration a case of a 27-year-old female with Crohn’s disease and whose mother also has Crohn’s disease (i.e., predisposing factor). While her disease is overall well-managed, about 6 months ago, she had an embarrassing episode of incontinence while at the gym (i.e., precipitating factor). Since then, she has limited her leaving the house and amount of regular exercise, which has led to significant weight gain, loneliness, increased anxiety, and overall made her more susceptible to additional flare ups of her disease and symptomology (i.e., perpetuating factors).

While much of the work in the area of psychogastroenterology (Rome Foundation, 2019) or behavioral gastroenterology (Jia et al., 2017) is correlational and somewhat new, much more work is needed in this area to determine the specific mechanisms by which behavioral and psychological factors affect or are affected by biological processes. As was outlined, behavioral and psychological factors could be implicated in the predisposing, precipitating, and perpetuating factors in gastrointestinal diseases. In the case of some of the more acute diseases, diet behavior could be targeted for treatment by a team of dieticians, physicians, and psychologists to encourage and facilitate meaningful behavior change. Some of the functional disorders may be perpetuated by behavioral, emotional, or cognitive factors as well, as has been demonstrated by the burgeoning literature demonstrating the benefits of cognitive behavior therapy (CBT; Kinsinger, 2017) and hypnosis for IBS (Palsson, 2015).

#### Dental, Oral, and Craniofacial Diseases

Oral disease accounts for some of the greatest worldwide burdens on health. Upward of 90% of schoolchildren in the world and a vast majority of adults experience dental caries (cavities) (World Health Organization, 2019). In the United States, nearly 50% of adults older than 30 years have some form of periodontitis (i.e., gum disease; Eke et al., 2015) which has been associated with heart disease, stroke, and could result in significant changes related to diet, health, quality of life, and well-being (Ng and Leung, 2006; Emami et al., 2013; Gargano and Hughes, 2014; Wright et al., 2017). In 2009, it was estimated that around 830,000 visits to an emergency department were associated with preventable dental disease (Pew Center on the States, 2012). Particularly in underserved communities, oral health has been described by the Surgeon General’s office as a “silent epidemic” (Department of Health and Human Services, 2000).

##### Predisposing factors

Much has been done to explore the potential long-standing, predisposing factors that can account for at least some of the variance in dental caries, periodontal disease, and other oral disease. In the case of dental caries, a number of genetic risk factors have been explored (Shaffer et al., 2013a). Genetics also have been associated with predisposing psychological factors in oral health. That is, some have demonstrated a predisposition toward pain sensitivity (e.g., *MC1R* gene) and its relation to poorer oral health (Randall et al., 2016, 2017a,b). Other long-standing factors such as SES, region, or access to dental insurance have been implicated in the utilization of dental services, which can influence oral health status (Chen et al., 2019). Others have shown the importance of familial transmission of dental care-related fear and anxiety (McNeil et al., 2019). All in all, a number of genetic, long-standing environmental, and family or cultural factors have shown to contribute to the development and maintenance of oral diseases.

##### Precipitating factors

More common oral diseases, such as dental caries and periodontal disease, do not develop overnight. A few examples are identified here of potential precipitating factors that may be contributing to the onset of oral health problems or pushing an individual over the oral health disease threshold. In some cases, there is more of a blend between the predisposing, precipitating, and perpetuating factors. Specifically, a behavior could sometimes facilitate the onset of dental decay or periodontal disease, but then a similar behavior is what perpetuates the problem. For example, stress, when experienced chronically has been identified as a risk factor for periodontal disease (Warren et al., 2014). Similarly, smoking has been associated with increases in periodontal inflammation (Nociti et al., 2014). Diet patterns such as drinking too much, too many sugar-sweetened beverages, or constant daily snacking on processed and sugary foods can elicit the development of dental decay (Marshall, 2013; Najeeb et al., 2016). Much like the application of the 3P-model to insomnia, it could be the acutely stressful times (e.g., loss of a job, working multiple jobs, having a newborn) that lead to a decrease in normally healthy oral health habits and hygiene, thus leading to disease.

As another example of a precipitating factor, women during pregnancy have been shown to have more periodontal inflammation (Figuero et al., 2013). There are a number of hypotheses why this occurs, but it mostly is attributed to a change in hormones during the perinatal time (Raber-Durlacher et al., 1994; Rai et al., 2008). The perinatal stressors are potential examples of precipitating factors that could lead to the development of behavior, cognitions, or emotions that eventually become perpetuating factors (González-Jaranay et al., 2017).

##### Perpetuating factors

After the onset of caries or periodontal disease, perpetuating factors become particularly relevant. That is, behavior plays a vital role in the alleviation of or the perpetuation of oral disease. Anecdotally, it is common to assume “if everyone just brushed and flossed, everything would be better.” A major perpetuating factor in this regard relates to oral health care utilization. Though the effectiveness of routine dental care is not yet clear (Davenport et al., 2003), many providers champion the regular attendance of routine cleanings and preventative appointments every 6 months to catch the development of caries or other disease early-on. Even when patients are in need of more urgent care, other behavioral factors also can be perpetuating by precluding individuals from regular attendance (Chen et al., 2019). For some, it may be the way they perceive dental care in a certain way or avoid care due to issues related to anxiety, fear, or pain (McNeil et al., 2011; McNeil and Randall, 2014; Kyle et al., 2016; Wright et al., 2017). While smoking can facilitate the onset of periodontal disease, it can perpetuate or worsen the disease as well (Nociti et al., 2014). It also could be that individuals lower the priority of their oral health care because of whatever else is going on in their life (Edwards et al., 2017).

##### Discussion of application to oral health

As is the case with all the diseases discussed here, biological, psychological, and social factors all play role in oral disease development and maintenance (Shaffer et al., 2013b). Consider a 49-year-old male raised in a rural Appalachian community with predisposing risk factors of poor access to fresh fruits and vegetables, and who worked in coal mining until about 2 years ago. In terms of precipitating factors, he has smoked since age 21 and after about age 30, stopped regular oral hygiene behavior such as brushing/flossing. He also stopped attending regular dental appointments, such as cleanings. Recently, he was seen by a dentist as part of a rural outreach by a state university’s dental school. Here, he was told that he has severe periodontal disease and is at risk of losing several teeth due to supportive bone loss. Some of the teeth might be saved if he were able to mitigate some of the potential perpetuating factors, including regularly accessing oral health care, improving his oral hygiene, and eliminating his smoking behavior. In this case, there is some legitimate overlap between some of the precipitating and perpetuating factors (e.g., poor oral hygiene can both precipitate and perpetuate oral disease such as periodontal disease). However, the 3P model accounts for the time course of the disease as well and helps clarify that behavior, thoughts, and emotion matter at all stages, but in particular in the perpetuation of oral disease due to the heavy influence of such factors in its chronicity. In the case of periodontal disease, care although it is unlikely that bone loss can be reversed (though interesting work seems promising in re-growing bone tissue; Tonelli et al., 2011), the disease progression can be halted through behavior modification, cognitive restructuring, and so on.

#### Heart Disease

Cardiovascular disease (CVD) is the leading cause of death for both men and women and is responsible for one in every four deaths each year in the United States and worldwide (Centers for Disease Control and Prevention, 2016). Sources of CVD include the vasculature, the myocardium, the heart’s electrical circuit, and congenital heart disease. Coronary artery disease (CAD), which is a disease of the blood vessels which supply the heart, is the most common type of CVD, and atherosclerosis (damage to the blood vessels), is a measure of CAD. Though there is variety in the possible diagnoses related to heart disease, our focus is on CAD, specifically the vasculature and the steady growth of arterial plaque overtime (Roger et al., 2012). Atherosclerosis is characterized by fatty plaque accumulation in the arteries that stiffens them, occluding blood flow to organs and tissues (Garcia and Khang-Loon, 1996). Not only is CAD deadly, but it is costly. Each year CAD costs the United States approximately $200 billion in health care, medicine, and lost work productivity (Centers for Disease Control and Prevention, 2016).

##### Predisposing factors

While CAD has long been understood as a disease perpetuated by poor health behaviors related to diet and/or exercise, CAD has underlying genetic risk factors as well, which is a predisposing factor for the disease. Approximately 40% of the risk for CAD is due to genetic factors (Marenberg et al., 1994). Specifically, high blood pressure and arteriosclerosis have strong genetic underpinnings (Lusis et al., 2004; Padmanabhan et al., 2012). While the heritability basis of CAD risk is known, researchers are still working to identify specific candidate genes which may serve as measurable markers of disease risk in human carriers (Biros et al., 2008; Lusis, 2012). This genetic risk is certainly nothing to overlook, as marathon runners and physically fit athletes, individuals who generally exercise and eat healthy, are also susceptible to CAD and related cardiac events (Noakes et al., 1977; Noakes, 1987).

In addition to inherited genes, there may be other family traits or learned behavior that are in a sense “heritable” as a result of shared environment with shared food access, learned diet and exercise patterns, and family modeling of appropriate stress coping mechanisms. Individuals who do not develop healthy eating habits, as well as those who do not engage in regular physical exercise are at an increased risk of carrying more body weight and therefore at greater risk for developing CAD when these behaviors are learned from a young age (i.e., the individuals do not know any different).

Additionally, there may be long-standing environmental factors that serve as predisposing factors for CAD. For example, certain geographic areas of the United States have higher rates of CAD than others. The states in the United States with highest CAD death rates (measured by coronary heart disease death rate per 1,00,000 people aged 35 and above, including both males and females) from the years 2014–2016 were Oklahoma, Arkansas, Tennessee, New York, West Virginia, and Michigan. On the other hand, the states with the lowest death rates included Minnesota, Hawaii, Oregon, Utah, and Colorado (Centers for Disease Control and Prevention, 2016).

Another well-known risk factor for CAD is SES (Schultz et al., 2018), which may be conceptualized as a predisposing factor. In high income countries, such as the United States, individuals living in low SES communities and neighborhoods are at an increased risk of developing CAD due to a number of psychosocial and behavioral risk factors, the latter of which are generally modifiable (Clark et al., 2009). Some studies have found that the specific facet of low SES that might be most related to CAD risk is low education status (Feldman et al., 1989; Winkleby et al., 1992). It would be important to consider patient SES when assessing for CAD risk factors, as not only may this mean that patients do not have the financial resources to receive preventative medical care or medical treatment, it may also be the case that these individuals require instruction and education regarding risk factors for CAD.

An additional, common predisposing factor related to CAD risk is related to chronic job stress. There is a robust literature which examines the relation between job stress and subsequent CAD risk and mortality, and variables related to perceived job stress include perceived job strain, high job demand and low perceived job control, and effort-reward imbalance (Kivimäki et al., 2002, 2006; Kershaw et al., 2016). Individuals typically do not begin a stressful job and experience negative cardiovascular effects right away. It is important to note that the effects of chronic job stress accumulate over years of exposure to the stressor (Chandola et al., 2006) and thus job stress is better conceptualized as a predisposing factor, as opposed to a more acute, precipitating factor.

A final example of an inherent predisposition to CAD lies in individual differences in risk for depression or experience of depressive symptoms. It is well-known that depression is prospectively associated with CAD onset (Musselman et al., 1998; Hare et al., 2013) and the American Heart Association has even added it as a risk factor for assessment (Lichtman et al., 2014). Depressed individuals are at greater risk of developing CAD than those who are not depressed, possibly through the effects of depression on motivation to engage in healthy behaviors (Musselman et al., 1998; Hare et al., 2013). That said, controlling for engagement in health behaviors, persons diagnosed with depression still possess a greater risk for CAD than non-depressed counterparts.

##### Precipitating factors

In considering risk factors contributing to CAD, it is apparent that the predisposing factors exert a strong influence and can at times be difficult to avoid. CAD risk increases as precipitating factors come into play. Precipitating factors which trigger emotional and psychological stress could include environmental and situational stressors of any kind. In the literature, several specific types of stressors have been linked with CAD risk and mortality. For example, one study in an urban setting found a significant association between neighborhood violent crime (an environmental factor), unemployment (a situational factor) and future risk of coronary heart disease over 1 year, such that increases in crime and unemployment rates led to increased CAD risk (Sundquist et al., 2006). Other situational triggers have been linked with CAD as well. For example, the occurrence of a major calamity such as an earthquake has been linked with short-term increases in risk factors related to CAD (higher heart rate, serum cholesterol levels, and serum triglyceride levels, etc.) (Trevisan et al., 1986; Kario et al., 2003). In addition, one study found a threefold increase in CAD related mortality following two earthquakes measuring 5.2 and 6.4 degrees on the Richter scale in Greece (Katsouyanni et al., 1986).

There is certainly a plethora of events that may increase one’s risk of CAD diagnosis (virtually any adverse environmental or situation factor imaginable), and the breadth of examples here serves to demonstrate the importance of considering the influence of many sorts of stressful experiences in disease care, especially since situations will likely vary largely between patients. Ultimately, as we will see, it is the perpetuating factors that one engages in as a consequence of these predisposing and precipitating factors that are most targetable.

##### Perpetuating factors

As the predisposing and precipitating factors may place one at an increased risk of CAD development, the perpetuating, behavioral factors are what may be targeted as a way to attenuate the deleterious effects of a CAD diagnosis. To cope with the predisposing and precipitating factors (including daily stressors), individuals may engage in a number of adverse health behaviors (Krueger and Chang, 2008). Imagine a single-working parent with a genetic predisposition to CVD who manages two jobs which pay the minimum wage. This individual is likely to experience issues related to job stress and low sense of job control. This parent is also likely so overworked that she does not feel she has the energy or time in her schedule to engage in regular exercise. Buying or preparing healthy meals may be time consuming and costly, and this family may need to rely on less expensive, more readily available prepackaged foods which are more likely to be high in salt, fat, and sugar.

For other individuals, adherence to treatment and medication regimens could be a perpetuating factor that maintains the risk for developing CAD or worsening symptoms. For some individuals, SES (which could be conceptualized as a predisposing or a precipitating factor), may actually contribute to this perpetuating factor of low medication adherence, as perhaps individuals who have lost a job or are living paycheck to paycheck cannot afford medication to treat or prevent CAD symptoms. For those who can afford medications as a preventative measure against CAD, adherence itself may be an issue. A 2012 meta-analysis found that adherence to CAD preventative medications is generally poor, and that these findings were not related to the type of drug prescribed (e.g., aspirin, ACE inhibitors, beta blockers, etc.), indicating that side effects were not a confounding variable (Naderi et al., 2012).

Additional perpetuating factors may be related to poor stress coping mechanisms. Though poor stress coping mechanisms were previously described as predisposing factors, these behaviors can also be conceptualized as perpetuating, as they may manifest as ways to cope with precipitating factors (e.g., job loss, grief), or with the stress of having a CAD diagnosis itself. Poor stress coping mechanisms include those substance use behaviors previously mentioned, including the negative health behaviors of smoking and drinking excessive alcohol. Individuals who rely on these coping mechanisms are thus at an even greater risk for subsequent CAD development (Centers for Disease Control and Prevention, 2016).

It is important to note that it is certainly possible to develop CAD without the perpetuating factors mentioned above, and the presence and specific type of perpetuating factors will vary from patient to patient. What would be important in an individual with no current perpetuating factors is a primary focus on the prevention and/or mitigation phases of the 3P model, depending on the “modifiability” of the relevant predisposing and precipitating factors (with genetic predisposition clearly being non-modifiable, but level of education being at least *somewhat* modifiable).

##### Discussion of application to heart disease

In the context of heart disease, the 3P model has clear applications for understanding relevant risk factors and related behaviors that contribute to an increased likelihood of CAD. As an example, imagine a 56-year old female with a family history of heart disease who has been working as an emergency room physician primarily on night shifts. This physician has relied most of her career on coffee and high calorie snack foods to get through her shifts, and poor sleep at inopportune times. Though she has had the option to switch to days, she enjoys the apparent freedom of having days off to attend family activities, which require further sacrifices in sleep. In general, genetic predisposition (such as family history in the example above) and learned health behaviors may initially predispose an individual to CAD. Stressful life events including job strain (e.g., working as an ER physician), job loss, loss of a loved one, and other traumatic experiences may precipitate the development of CAD. What maintains an individual’s heightened level of risk are adverse health behaviors (e.g., poor diet and sleep habits) related to CAD prevention and a lack of healthy coping mechanisms.

In thinking of interventions to assist our emergency room physician, an area for behavioral change would be for a physician to educate her about the health risks associated with working night shift, and to encourage her to switch to day shifts. Switching to day shifts would allow her to settle into a more consistent nightly sleep schedule and eliminate nighttime eating, something that was necessary during overnight shifts but has been linked to poor health. If switching her shift is not a feasible option, dietary counseling on healthy snack options during her shift would be beneficial. Working with this individual to problem-solve and make changes where possible will help to reduce risky health behaviors that only exacerbate her disease risk.

It is important to consider that although the 3P model suggests three separate types of risk factors, when applied to CAD, these three categories are not mutually exclusive. That is, one individual’s predisposing factor may function as another individual’s precipitating factor. Or, for example, poor coping mechanisms may first act as a predisposing factor, but could later manifest as a perpetuating factor. From a clinical psychology perspective, it is necessary to identify antecedent factors that may put one at an increased risk of disease development. As evidenced by this model, however, it is just as important to consider *maintaining* factors in establishing a set of behaviors, behaviors which themselves have both antecedent and consequential effects. In this case, it is not the parameter itself but its function. To expand on the example given above, it is not necessarily important whether the poor coping mechanism is overeating, smoking, or drinking, what matters is the *function* of the behavior, which is to cope. These are important aspects of disease development for therapists to consider.

An additional issue to consider is how to use this model to explain sudden heart attacks or other health concerns in individuals who do engage in adaptive health behaviors by eating nutritious foods and regularly exercising. For these individuals, perhaps their genetic predisposition is what determines their ultimate outcome.

The 3P model advances the conceptualization of CAD as more than just a biological disease resulting from poor genetic luck or from purely internal, physiological factors. The model considers both internal factors (e.g., genetic) and external factors (e.g., life events, stressors, and behaviors) that all culminate and contribute to one’s overall risk of disease development. While it is important to attend check-ups and monitor blood pressure, cholesterol levels, and the like, it is just as important to consider the breadth of precipitating and perpetuating factors that psychologists may target in order to treat, mitigate, and prevent subsequent CAD development.

## Discussion and Implications for the Future

The primary aim of this paper was to provide a framework and conceptualization of how psychological considerations can be integrated into an overall multidisciplinary disease model of practice and research. The 3P model enhances the utility of and ameliorates some limitations of the other models in this area, such as the biopsychosocial model. In particular, the 3P model includes a time course and expands on *how* disease develops and is maintained. It also aids in demonstrating the importance of integrated perspectives when studying, preventing, mitigating, or treating a variety of disease states and conditions.

In terms of research, scientists might utilize the 3P disease model to conceptualize and identify actual predisposing, precipitating, and perpetuating factors for these and many other diseases. In this way, the model provides a framework for understanding where each discovered component “fits in,” or the mechanisms in the etiology of various disease states. Relatedly, the model encourages future researchers to think about “What *are* the predisposing, precipitating, and perpetuating factors of x, y, or z disease?,” and “In what ways can each be treated by a psychologist or with a psychologist integrated into another team such as dieticians, physicians, physical therapists, dentists, etc.?” Future research could focus on clinical utility of teams conceptualizing disease in terms of the 3P model and examine patient satisfaction, disease prevention, or other outcomes, much like Bolton (2014) suggests with another proposed model. The 3P model, however, adds to the practicality of implementation to multiple diseases based on its prior theoretical application to insomnia. Ideally, others will utilize and adapt the framework of 3P disease model presented here to many other diseases, including but not limited to type II diabetes, cystic fibrosis, asthma, stroke recovery, or cancer. Perhaps gains in understanding of these disease states will translate to effective interventions with more conceptually difficult diseases, such as Alzheimer’s or Parkinson’s disease.

The 3P model also facilitates potential explanations for current and future clinical applications to be developed, studied, and implemented. Implications for potential interventions also are displayed in Figure 1 as possible ways of suppressing any predisposing, precipitating, and perpetuating factors. Interventions at the premorbid stage of disease development, whether targeted for biological, socio-environmental, or psychological factors, would be considered preventative. For example, an intervention that targets the prevention of oral disease in an environment such as rural Appalachia could focus on the broader societal, economic, environmental, or even cultural factors that serve as long-standing predisposing factors of oral health status. For individuals living in this area, financial concerns as well as limited access to dental care may function as predisposing factors that interfere with proper care. Interventions targeted in the acute and early stages of disease development would be used to mitigate the potential for chronic problems related to the disease. For example, offering psychoeducation, physical therapy, and social support to individuals who have been injured in an automobile accident may prevent any subsequent pain problems from becoming a chronic issue. Also, interventions targeted to the acute and chronic phases of disease development could be seen as more traditional “treatment” as it is understood today (i.e., a response to the manifestation of a disease-like state). This approach might include cognitive behavior therapy for gastrointestinal diseases or behavior modification in addition to medical interventions for heart disease patients.

In considering ways in which various diseases manifest over time – be it predisposing, precipitating, or a perpetuating factors – common issues related to maladaptive health behavior or cognitions arise. Behavior is complex, multi-faceted, and underlies many chronic diseases. A crucial component of initiating change is understanding barriers and resistance. In clinical settings, the 3P model may contribute a unique and fresh perspective to behavioral concerns in the prevention of disease (i.e., at the predisposing or precipitating stage of disease development) and through involvement in patient treatment and interventions (i.e., at the perpetuating stage of disease maintenance). Interventions by health care providers might include providing assessment of psychological risk factors, making behavioral observations and providing behavioral interventions to individuals and families, and evaluating the effectiveness of various psychological based treatments on disease management and prevention.

The 3P model of health moves healthcare toward prevention or mitigation of diseases, rather than a traditional treatment perspective alone. Overall, the 3P model clarifies and provides a framework for providers to explicate and ameliorate disease etiology and maintenance processes.

## Conclusion

The 3P model provides a conceptualization of health conditions and disease states based on predisposing, precipitating, and perpetuating factors. Both the development and maintenance of health problems are addressed, allowing for a longitudinal understanding. Originally applied to insomnia, the 3P model here is applied to chronic pain, gastrointestinal disease, dental, oral, and craniofacial diseases, and CAD. The model provides a framework for a broad array of other health conditions and disease states as well, providing a basis for an integrative approach to understanding and treating health problems through an enhanced biopsychosocial perspective.

## Author Contributions

CW initiated, directed, and aided in the conceptualization, writing, revising, editing, and submitting of the manuscript. AT and AB aided in the conceptualization, writing, revising, and editing of the manuscript. SS, KL, and DM aided by giving overall feedback about the conceptualization and writing, revising, and editing of the manuscript.

## Conflict of Interest

The authors declare that the research was conducted in the absence of any commercial or financial relationships that could be construed as a potential conflict of interest.
